# FINANCIAL HARDSHIP AND INCOME SECURITY AND MENTAL HEALTH AMONG OLDER WORKERS: MULTI-CHANNEL SEQUENCE APPROACH

**DOI:** 10.1093/geroni/igac059.3061

**Published:** 2022-12-20

**Authors:** Oejin Shin, Sojung Park, Eunsun Kwon, Byeongju Ryu

**Affiliations:** Illinois State University, Savoy, Illinois, United States; Washington University in Saint Louis, Saint Louis, Missouri, United States; Fairleigh Dickinson University, Florham Park, New Jersey, United States; Washington University in St. Louis, St. Louis, Missouri, United States

## Abstract

Drawing on the life course perspective and multi-channel sequence approach, this research identifies long-term financial hardship and income security patterns among older adults and examines their relationship with depressive symptoms. Data were from the 2010 to 2018 HRS with 3,077 older workers aged 62–71 in 2010. Four clusters of key sequences were identified: 1) High food and low medical insecurities with private pension plans (43%); 2) Moderate food and low medical insecurities with gradually acquired private pension plans (13%); 3) High food and low medical insecurities with both private and employment-based pension plans (40%); 4) High food insecurity with employment-based pension plans(3%). Regarding the association with depressive symptoms, a group of people who experienced high food and low medical insecurities with private pension plans (b= 0.14, p < 0.1) and those who experienced moderate food and low medical insecurities with gradually acquired private pension plans (b= 0.32, p < 0.01) were more likely to have depressive symptoms compared to those high food and low medical insecurities with both private and employment-based pension plans. These findings highlight the heterogeneity of long-term patterns of financial hardships and patterns of income security, including the limited accessibility for a pension plan through their occupation and that an individual pension plan may not be sufficient to ensure mental health in later life. The results imply the importance of dual security of private and employment-based pensions on their mental health and provide important insights for future tailored income support programs for older adults.

